# Femtosecond Laser in Anterior Segment Surgery

**DOI:** 10.1155/2021/9825254

**Published:** 2021-08-27

**Authors:** Sang Beom Han, Jodhbir S. Mehta, Yu-Chi Liu, Karim Mohamed Noriega

**Affiliations:** ^1^Department of Ophthalmology, Kangwon National University School of Medicine, Kangwon National University Hospital, Chuncheon 24289, Republic of Korea; ^2^Singapore National Eye Centre, Singapore; ^3^Singapore Eye Research Institute, Singapore; ^4^Department of Ophthalmology, Yong Loo Lin School of Medicine, National University of Singapore, Singapore; ^5^Department of Ophthalmology, University Hospital, Faculty of Medicine, Autonomous University of Nuevo Leon, Monterrey, Mexico

The application of femtosecond lasers in ophthalmology has enabled precise and reproducible tissue cutting, which is expected to improve the outcomes of surgical treatment, particularly in anterior segment surgeries such as cornea, cataract, and refractive surgery.

As we have introduced in the call for papers for this Special Issue, the articles published cover topics focused on the application of femtosecond lasers in anterior segment surgery, including the development of the technologies behind femtosecond lasers and their application in ophthalmic surgery, the efficacy and safety of anterior segment surgery using femtosecond lasers, and imaging techniques associated with the application of femtosecond lasers.

Femtosecond lasers have been increasingly used in cornea and refractive surgery. The laser can be used to create customized trephination edges for deep anterior lamellar keratoplasty and penetrating keratoplasty and for allowing ultrathin cut for Descemet stripping automated anterior lamellar keratoplasty. It can also be helpful for improving the consistency and reproducibility of refractive surgery, including laser-assisted in situ keratomileusis (LASIK), small incision lenticule extraction (SMILE), intrastromal corneal ring segment implantation, and astigmatic keratotomy.

Femtosecond laser cataract surgery (FLACS), in which a femtosecond laser is employed for corneal incision, anterior capsulotomy, lens fragmentation, and liquefaction, is also expected to improve the reproducibility and safety of cataract surgery. In this Special Issue, the authors contributed 10 original papers and one review article regarding the use of femtosecond lasers in anterior segment surgery.

The authors have reported the results of their research on various topics related to femtosecond lasers in anterior segment surgery: (1) extrusion of femtosecond laser-implanted intrastromal corneal ring segments in keratoconic eyes: prevalence, risk factors, and clinical outcomes; (2) clinical observation of silicon hydrogel contact lens fitted immediately after SMILE; (3) preliminary results of a novel standardized technique of femtosecond laser-assisted deep anterior lamellar keratoplasty for keratoconus; (4) long-term evaluation of capsulotomy shape and posterior capsule opacification after low-energy bimanual femtosecond laser-assisted cataract surgery; (5) anatomical and visual outcomes after LASIK performed in myopic eyes with the WaveLight® refractive suite (Alcon® Laboratories Inc., USA); (6) correlation analysis of refractive and visual quality after wavefront-optimized laser in situ keratomileusis for 50% and 100% angle kappa compensation; (7) comparison of femtosecond laser-assisted cataract surgery and conventional phacoemulsification in shallow anterior chambers and glaucoma; (8) characteristics of facial asymmetry in congenital superior oblique palsy according to trochlear nerve absence; (9) a modified femtosecond laser technique for anterior capsule contraction syndrome; and (10) evaluation of astigmatic correction using a vector analysis after combined femtosecond laser-assisted phacoemulsification and intrastromal arcuate keratotomy. This Special Issue also includes one review article on the application of femtosecond lasers in anterior segment surgery.

We believe these papers will provide readers with valuable information on the application of femtosecond lasers in anterior segment surgery and new ideas for research on related topics.

